# Role of School Bullying Involvement in Depression, Anxiety, Suicidality, and Low Self-Esteem Among Adolescents With High-Functioning Autism Spectrum Disorder

**DOI:** 10.3389/fpsyt.2020.00009

**Published:** 2020-01-31

**Authors:** Wen-Jiun Chou, Peng-Wei Wang, Ray C. Hsiao, Huei-Fan Hu, Cheng-Fang Yen

**Affiliations:** ^1^ Department of Child and Adolescent Psychiatry, Chang Gung Memorial Hospital, Kaohsiung Medical Center and College of Medicine, Chang Gung University, Taoyuan, Taiwan; ^2^ Department of Psychiatry, Kaohsiung Medical University Hospital, Kaohsiung, Taiwan; ^3^ Department of Psychiatry, School of Medicine, and Graduate Institute of Medicine, College of Medicine, Kaohsiung Medical University, Kaohsiung, Taiwan; ^4^ Department of Psychiatry and Behavioral Sciences, University of Washington School of Medicine, Seattle, WA, United States; ^5^ Department of Psychiatry, Children’s Hospital and Regional Medical Center, Seattle, WA, United States; ^6^ Department of Psychiatry, Tainan Municipal Hospital (Managed by Show Chwan Medical Care Corporation), Tainan, Taiwan

**Keywords:** autism spectrum disorder, school bullying, depression, anxiety, suicidality, self-esteem

## Abstract

This study aimed to compare the severities of psychopathologies and the level of self-esteem among 219 adolescents with high-functioning autism spectrum disorder (ASD) who were classified into pure perpetrators, pure victims, perpetrator-victims, and neutrals in Taiwan. The experiences of school bullying involvement in the previous 1 year were measured using the Chinese version of the School Bullying Experience Questionnaire. The severities of depression, anxiety, and suicidality were measured using the Center for Epidemiological Studies Depression Scale, the Multidimensional Anxiety Scale for Children, and the 5-item questionnaire from the epidemiological version of the Kiddie Schedule for Affective Disorders and Schizophrenia, respectively. The level of self-esteem was measured using the Rosenberg Self-Esteem Scale. The results indicated that compared with the self-reported neutrals, the self-reported perpetrator-victims and pure victims had more severe depression and anxiety. No difference in depression, suicidality, anxiety, and self-esteem was found among the four groups of various parent-reported bullying involvement experiences. Mental health problems in adolescents with ASD who experienced bullying victimization should be routinely surveyed.

## Introduction

The true extent of school bullying in adolescents with autism spectrum disorder (ASD) remains an underresearched area (1). This research gap is a concern because adolescents with ASD may be vulnerable to bullying given their difficulties in social relationships that are hallmarks of their characteristics (1, 2). A meta-analysis found that the estimated prevalence for school bullying perpetration, victimization, and both school bullying perpetration and victimization among youth with ASD was 10%, 44%, and 16%, respectively (3). Moreover, school-aged youth with ASD are at a greater risk of school victimization than their typically developing peers (3). Studies have proposed several possible etiologies to account for the high risk of bullying victimization in adolescents with ASD, including communication problems (4), fewer friendships (5), stereotyped behavior and interests (6), and aggressive behaviors (7). A recent study based on the perspectives of adolescents and their parents found that involvement in bullying is one of major daily living stressors experienced by the adolescents with ASD, as well as that the experience of bullying involvement was resulted from the core symptoms and characteristic behaviors of ASD (8). Studies examining the experiences of adolescents with ASD could help develop individualized behavioral interventions to reduce the bullying-related stress for adolescents with ASD.

Studies on children and adolescents from the general population have found that compared with neutrals, bullying victims have significant risks of depression (9), anxiety (10), and suicidal ideation and suicide attempts (11). In recent years, an increasing number of studies have assessed the association of bullying involvement with mental health problems in children and adolescents with ASD (12. A longitudinal study found that social communication impairments at the age of 10 years were associated with depression at the age of 18 years, and bullying victimization explained a substantial proportion of this risk (13 ). Parent-reported bullying victimization was significantly associated with anxiety symptoms in youth with ASD (14, 15). Children and adolescents with ASD who were victims of school bullying had a higher risk of suicidality than did those with ASD who were not the victims of school bullying (16). These results of previous studies indicate that school bullying prevention programs are warranted, and that intervention programs should be implemented for children and adolescents with ASD who have experienced bullying victimization to prevent the consequences of mental health problems (14).

Several important concerns regarding bullying involvement and mental health in adolescents with ASD warrant further study. First, most of attention was drawn to the mental health problems of bullying victims; however, research has found that bullying perpetration was associated with depression (9) and suicidal ideation and suicide attempts in the general adolescent population (11). Whether the risk of mental health problems increases in adolescents with ASD who perpetrated bullying on peers is not known.

Second, the individuals can be categorized into four groups based on their roles in bullying. Those who bullied others but were not bullied by others are pure perpetrators. Those who were bullied by others but did not bully others are pure victims. Those who were bullied by others and also bullied others are perpetrator-victims. Those who neither bullied others nor were bullied by others are the neutrals. Perpetrator-victims have been found to be the most troubled among these four groups (17). Research on the adolescent population has found that perpetrator-victims have the higher risks of depression and suicidality than did pure perpetrators and pure victims (18). Research has found that perpetrator-victims have a unique formation process and role transition (19); they also have the stress coping strategies different with pure victims and pure perpetrators (20, 21). Further study is needed to examine whether the differences in the risk of comorbid mental health problems also exist among adolescents with ASD who are perpetrator-victims, pure victims, and pure perpetrators. The results of the studies can provide knowledge to develop prevention and intervention programs for mental health problems in bullying perpetrator-victims with ASD.

Third, because of different awareness, definitions, and attributions, self-reported bullying involvement by adolescents with ASD may be different from that reported by their parents (3). Given that the sources of information may influence what the clinicians know about ASD adolescents’ bullying involvement, additional studies are required to examine whether the association between bullying involvement and mental health problems varies according to the various sources of information on bullying involvement.

The present study examined the differences in the levels of depression, anxiety, suicidality, and self-esteem among adolescents with high-functioning ASD categorized according to various experiences of school bullying involvement: those without any involvement in bullying (neutrals), pure victims, pure perpetrators, and perpetrator-victims. We have three hypotheses. First, based on the results of previous studies on ASD youth with bullying victimization (14–16) and general adolescent population with bullying perpetration (9, 11), we hypothesized that in adolescents with ASD, pure victims, pure perpetrators, and perpetrator-victims have more severe depression, anxiety, and suicidality and lower self-esteem than do neutrals. Second, based on the results of previous studies on general adolescent population (17, 18), we hypothesized that in adolescents with ASD, perpetrator-victims have more severe depression, anxiety, and suicidality and lower self-esteem than do pure victims and pure perpetrators. Third, given that adolescents with ASD may have different awareness, definitions and attributions of the experiences of bullying involvement compared with their parents (3), we hypothesized that the association of adolescent-reported bullying involvement with mental health problems is different from that of parent-reported bullying involvement with mental health problems.

## Materials and Methods

### Participants

The study participants were enrolled from five child psychiatry outpatient clinics in Taiwan; three clinics were at university-affiliated teaching hospitals, one was at a regional teaching hospital, and one was an outpatient clinic specific for child psychiatry. Patients enrolled in the Taiwan National Health Insurance program are allowed to visit the outpatient clinics of teaching hospitals without referrals from general practitioners. Therefore, the adolescents enrolled from these five child psychiatry outpatient clinics in the present study are representative of similar-age populations in Taiwan. We recruited adolescents who were at the age of 11–18 years and had high-functioning ASD consecutively to this study between August 2013 and July 2016. The diagnosis of ASD was determined according to the *Diagnostic and Statistical Manual of Mental Disorders, Fifth Edition* [DSM-5; (22)]. The criteria of “high-functioning” were that the adolescents had verbal communication ability with a full-scale intelligence quotient (FSIQ) of >80 as determined using the Wechsler Intelligence Scale for Children, Fourth Edition (23). Parents who had schizophrenia, bipolar disorder, intellectual disability, or any cognitive deficit that resulted in significant communication difficulties were excluded. A total of 228 adolescents with high-functioning ASD who complied with the criteria above were approached. Child psychiatrists explained the purpose and procedure of this study to adolescents and their parents and invited them into this study. Of them, 219 (96.1%) adolescents and their parents agreed to participate in this study and were interviewed by research assistants using the research questionnaire.

### Measures

#### Chinese version of the School Bullying Experience Questionnaire (C-SBEQ)

 The self-reported C-SBEQ contains 16 items evaluated on a 4-point Likert scale from 0 (never) to 3 (all the time) (24). This scale is composed of two 8-item subscales that evaluate whether an adolescent is a bullying victim (items 1–8, including social exclusion, being called a mean nickname, being spoken ill of, being beaten up; being forced to do work; and having money, school supplies, and snacks taken away) and a bullying perpetrator (items 9–16) in the previous 1 year. The participants who scored 2 or 3 on any item among items 1–8 and 9–16 by themselves and their parents were identified as self-reported and parent-reported bullying victims and perpetrators, respectively. The participants were further categorized into pure perpetrators, pure victims, perpetrator-victims, and neutrals. The results of factor analysis in a previous study on Taiwanese adolescents found that the values of indices met goodness-of-fit standards and support the factor structure of the C-SBEQ. The Cronbach α of the subscales for bullying victimization and perpetration on the C-SBEQ was 0.727 and 0.753, respectively, indicating acceptable internal-consistency reliability. The average intraclass correlation coefficients (ICC) for the subscales on the C-SBEQ was from 0.788 to 0.813, which indicated moderate to high levels of 1-month test–retest reliability. The consistency between the self-reported and nominated victims or perpetrators of bullying was examined using generalized kappa coefficients (κ). The κ coefficients for being victims and perpetrators of bullying were 0.547 and 0.478, respectively, which indicated moderate agreement between self-reported and nominated victims or perpetrators of bullying and supported the validity of the cutoff (≧2) to discriminating victims and perpetrators from nonvictims and nonperpetrators, respectively.

#### Taiwanese version of the Center for Epidemiological Studies Depression Scale (T-CES-D)

The self-reported T-CES-D comprises 20 items evaluated on a 4-point Likert scale from 1 (rarely or none of the time) to 4 (most or all of the time) that assess the frequency of depressive symptoms in the recent 1 month (25, 26). A higher total score on the T-CES-D indicates more severe depression. A previous study on Taiwanese adolescents supported the discriminatory validities of the T-CES-D by discriminating those with and without major depressive disorder and dysthymic disorder (27). Previous studies on Taiwanese adolescents have confirmed the congruent validity of the T-CES-D by establishing its significant correlations with suicide tendency (28), insomnia (29), and poor peer relationships (30). In the present study, Cronbach’s α of the T-CES-D was 0.88.

#### Taiwanese version of the Multidimensional Anxiety Scale for Children (MASC-T)

The self-reported MASC-T comprises 39 items evaluated on a 4-point Likert scale that assess the level of anxiety symptoms in the recent 1 month (31, 32). A higher total score on the MASC-T indicates more severe anxiety symptoms. The results of a previous study proved that the MASC-T has acceptable internal-consistency reliability (Cronbach’s alpha coefficients ranged from 0.871 to 0.895 in boys and 0.880 to 0.894 in girls), 1-month test–retest reliability (*p* values of single and average ICC < 0.001) and validity (*p* value of *t* test discriminating adolescents with anxiety disorder from those without anxiety disorders < 0.001) (32). In the present study, Cronbach’s α for the MASC-T was 0.88.

#### Suicidality

The Suicidality module of the epidemiological version of the Kiddie Schedule for Affective Disorders and Schizophrenia (33) contains five self-reported items elicited a yes or no response that assess the occurrence of suicide attempts and four forms of suicidal ideation in the preceding year (28). In a previous study, Cohen’s kappa coefficient of agreement (κ) between adolescents’ self-reported suicidality and their parents’ reports was 0.541 (*p* < 0.001) (28). A higher total number of yes responses for the items indicate a higher risk of suicidality. In the present study, Cronbach’s α for the questionnaire on suicidality was 0.79.

#### Rosenberg Self-Esteem Scale (RSES)

The self-reported RSES comprises 10 items evaluated on a 4-point Likert scale that assess current self-esteem. This scale has acceptable reliability and construct validity (34). A higher total score on the RSES indicates a high level of self-esteem. A previous study on Taiwanese adolescents with ADHD found that the RSES had acceptable internal-consistency reliability (Cronbach’s alpha coefficient = 0.861) and congruent validity by establishing negatively associations of RSES score with depressive (*p* < 0.001) and anxiety (*p* < 0.001) symptoms (35). In the present study, Cronbach’s α was 0.86.

#### Chinese Social Responsiveness Scale (SRS)

The parent-reported Chinese SRS contains 25 items evaluated on a 4-point Likert scale that assess adolescents’ reciprocal social behaviors. A higher total score indicates greater impairments of social responsiveness. Research has found that the SRS effectively distinguish between children and adolescents with and without ASD (36, 37).

#### Short form of the Swanson, Nolan, and Pelham Version IV Scale (SNAP-IV)-Chinese version

The parent-reported short form of the SNAP-IV-Chinese version comprises 26 items rated on a 4-point Likert scale from 0 (not at all) to 3 (very much) that assess adolescents’ inattention, hyperactivity/impulsivity, and oppositional symptoms based on the DSM Fourth Edition (DSM-IV) criteria of attention-deficit/hyperactivity disorder (ADHD) (38, 39). Higher total scores on the subscales indicate greater ADHD and oppositional symptoms. In the present study, Cronbach’s α values of the inattention, hyperactivity/impulsivity, and oppositional subscales were 0.91, 0.91, and 0.92, respectively.

### Procedure

The adolescents with high-functioning ASD and their parents were invited to complete the research questionnaires. Two master-degree research assistants conducted an individual interview to collect data on the adolescents’ self-reported school bullying experiences, depression, anxiety suicidality, and self-esteem. Before conducting the research interviews, research assistants received comprehensive training in the application of research questionnaires. The principal investigator (CFY) introduced the contents of research questionnaires to and had the discussion with research assistants. Then each research assistant conducted a research interview with an adolescent with high-functioning ASD under supervision of the principal investigator (CFY) and then received feedback for modification of the interview. Meanwhile, research assistants were requested to interview in accordance with the research questionnaire to make sure the inter-interviewer reliability. Research assistants introduced all questionnaires with standard guidance. For example, research assistants said when they started interviews based on the C-SBEQ: “I would like to learn about your feelings and the behaviors of your classmates. Your answers will help me to find out how to make things better at school for children. Please feel free to ask me for any other reason.” All interviews were conducted individually in the interview rooms of outpatient clinics. Research assistants’ performance during the interviews was supervised regularly to assess fidelity. The parents completed the C-SBEQ, Chinese SRS, and short form of SNAP-IV. Data analysis was performed using SPSS 20.0 statistical software (SPSS Inc., Chicago, IL, USA).

### Statistical Analysis

Sex, age, social communication deficits, inattention, hyperactivity/impulsivity, and opposition among the groups of various self-reported and parent-reported bullying involvement (pure victims, pure perpetrators, perpetrator-victims, and the neutrals) were compared using chi-square test and analysis of variance (ANOVA). Because of multiple comparisons, a *p*-value of <0.005 (0.05/10) was considered statistically significant. Depression, anxiety, suicidality, and self-esteem among the groups of various self-reported and parent-reported bullying involvement were compared using multivariate analysis of variance (MANOVA). A *p*-value of <0.05 was considered statistically significant. The differences in the levels of depression, anxiety, suicidality, and self-esteem among four groups of adolescents with various self-reported and parent-reported bullying experiences of bullying involvement were further examined using multivariate analysis of covariance (MANCOVA), in which the effects of sex, age, social communication deficits, inattention, hyperactivity/impulsivity, and opposition were controlled for. Weight least square was used to adjust unequal variance for MANCOVA.

### Ethics

All adolescents and their parents provided written informed consent. Parents also provided written informed consent to agree their children participating into this study. The study was conducted in accordance with the Declaration of Helsinki, and the protocol was approved by the Ethics Committee of Kaohsiung Medical University Hospital (KMUHIRB-20120084).

## Results

In total, 219 (192 boys and 27 girls) adolescents and their parents participated in this study. Their mean age was 13.7 years [standard deviation (SD) = 2.1 years]. Their mean FSIQ was 95.4 (SD = 9.6). All participants had the ability to communicate verbally with others without any difficulty based on their parents’ observation and clinical observation. Their mean score of social communication deficits on the SRS was 104.6 (SD = 29.2; range: 27–179). The means, standard deviations, and correlation matrices of the measured variables are shown in **Table 1**. The results revealed significant correlations among the measured variables.

**Table 1:** The correlation matrix of measured variables.

	Mean (SD)	1	2	3	4	5	6	7	8	9	10
1. Bullying victimization	2.7 (2.8)	1	0.482**	0.173	0.118	0.120	0.025	0.335**	0.379**	0.238**	−0.140
2. Bullying perpetration	1.8 (2.4)		1	0.143	0.096	0.200*	0.195*	0.305**	0.333**	0.220^*^	−0.171
3. Social communication deficits	154.5 (27.2)			1	0.663**	0.531**	0.388**	0.197*	0.150	0.197^*^	−0.137
4. Inattention	14.7 (6.5)				1	0.671**	0.584**	0.216*	0.071	0.148	−0.161
5. Hyperactivity/impulsivity	10.0 (6.7)					1	0.661**	0.098	0.062	0.126	−0.053
6. Opposition	10.7 (6.3)						1	0.205*	0.079	0.216^*^	−0.189
7. Depression	14.6 (10.1)							1	0.617**	0.407**	−0.652^**^
8. Anxiety	35.2 (16.8)								1	0.264**	−0.356^**^
9. Suicidality	0.4 (1.0)									1	−0.372^**^
10. Self-esteem	19.9 (6.1)										1

*p < 0.005; **p < 0.001.

**Tables 2** and **3** present the ratios of the self-reported and parent-reported pure victims, pure perpetrators, perpetrator-victims, and the neutrals with high-functioning ASD. The rates of the self-reported neutrals, pure victims, pure perpetrators, and perpetrator-victims were 63.9%, 17.8%, 9.1%, and 9.1%, respectively. The rates of the parent-reported neutrals, pure victims, pure perpetrators, and perpetrator-victims were 41.6%, 23.7%, 5.9%, and 28.8%, respectively. The ASD adolescents self-reported a higher rate of being pure perpetrators than that reported by the parents (9.1% vs. 5.9%), whereas the parents reported higher rates of being pure victims (23.7% vs. 17.8%) and perpetrator-victims (28.8% vs. 9.1%) than those reported by the ASD adolescents. Low agreement was found between self-reported and parent-reported bullying involvement (kappa = 0.101). Only 10 (4.6%), 1 (0.5%), and 8 (3.7%) ASD adolescents were simultaneous self-reported and parent-reported pure victims, pure perpetrators, and perpetrator-victims, respectively.

**Table 2:** Sex, age, social communication deficits, inattention, hyperactivity/impulsivity, opposition, depression, anxiety, suicidality, and self-esteem among the groups of various self-reported bullying involvement (*N* = 219).

	Self-reported bullying involvement						*Post hoc* comparison
	Neutrals (*n* = 140; 63.9%)	Pure victims (*n* = 39; 17.8%)	Pure perpetrators (*n* = 20; 9.1%)	Perpetrator-victims (*n* = 20; 9.1%)	χ^2^ or *F*	*p*	
Sex^a^							
Girls (*n* = 27)	17 (63)	4 (14.8)	2 (7.4)	4 (14.8)	1.349	0.718	
Boys (*n* = 192)	123 (64.1)	35 (18.2)	18 (9.4)	16 (8.3)			
Age (years)^b^	13.6 (2.1)	14.1 (2.0)	13.9 (1.7)	13.6 (2.2)	0.837	0.475	
Social communication deficits^b,c^	150.6 (25.9)	158.2 (32.3)	163.0 (29.3)	165.6 (18.2)	3.029	0.030	
Inattention^b,c^	14.3 (6.3)	14.3 (7.5)	17.5 (6.1)	15.5 (6.2)	1.562	0.200	
Hyperactivity/impulsivity^b,c^	9.2 (6.2)	9.9 (6.1)	12.9 (8.2)	12.3 (8.5)	2.676	0.048	
Opposition^b,c^	10.5 (5.9)	10.4 (6.5)	12.5 (6.9)	11.2 (7.6)	0.649	0.584	
Depression^b,d^	12.5 (8.9)	18.2 (11.5)	17.0 (8.8)	20.4 (12.1)	3.708	<0.001	Perpetrator-victims > Neutrals Pure victims > Neutrals
Anxiety^b,d^	31.1 (15.8)	40.6 (15.9)	40.7 (14.9)	47.9 (16.8)			Perpetrator-victims > Neutrals Pure victims > Neutrals
Suicidality^b,d^	0.2 (0.7)	0.7 (1.3)	1.0 (1.6)	1.0 (1.3)			
Self-esteem^b,d^	20.4 (6.0)	19.2 (6.0)7	20.6 (4.8)	17.4 (8.0)			

^a^n (%); ^b^mean (SD); ^c^data were analyzed by ANOVA; ^d^data were analyzed by MANOVA.

**Table 3:** Sex, age, social communication deficits, inattention, hyperactivity/impulsivity, opposition, depression, anxiety, suicidality, and self-esteem among the groups of various parent-reported bullying involvement (*N* = 219).

	Parent-reported bullying involvement						*Post hoc* comparison
	Neutrals (*n* = 91; 41.6%)	Pure victims (*n* = 52; 23.7%)	Pure perpetrators (*n* = 13; 5.9%)	Perpetrator-victims (*n* = 63; 28.8%)	χ^2^ or *F*	*p*	
Sex^a^							
Girls (*n* = 27)	11 (40.7)	8 (70.4)	0	8 (70.4)	2.290	0.514	
Boys (*n* = 192)	80 (41.7)	44 (22.9)	13 (6.8)	55 (28.6)			
Age (years)^b^	13.3 (2.1)	13.6 (2.2)	14.4 (1.8)	14.2 (1.9)	3.147	0.026	
Social communication deficits^b,c^	140.1 (27.8)	159.6 (22.5)	161.0 (20.5)	169. (20.6)	19.681	<0.001	Perpetrator-victims > Neutrals Pure victims > Neutrals Pure perpetrators > Neutrals
Inattention^b,c^	11.9 (6.2)	15.5 (5.7)	15.5 (5.3)	17.7 (6.3)	11.850	<0.001	Perpetrator-victims > Neutrals Pure victims > Neutrals
Hyperactivity/impulsivity^b,c^	7.2 (5.7)	11.4 (6.4)	10.8 (7.6)	12.5 (6.9)	10.203	<0.001	Perpetrator-victims > Neutrals Pure victims > Neutrals
Opposition^b^	8.9 (1.0)	9.5 (6.3)	13.4 (7.1)	13.9 (6.5)	10.453	<0.001	Perpetrator-victims > Neutrals
Depression^b,d^	12.7 (9.6)	14.0 (8.5)	16.7 (9.8)	17.5 (11.4)	2.067	0.018	Perpetrator-victims > Neutrals
Anxiety^b,d^	31.3 (16.5)	35.3 (16.9)	39.5 (21.6)	40.0 (14.9)			Perpetrator-victims > Neutrals
Suicidality^b,d^	0.4 (1.0)	0.3 (0.7)	0.2 (0.6)	0.7 (1.3)			
Self-esteem^b,d^	20.3 (6.2)	21.1 (5.9)	17.0 (5.4)	19.1 (6.2)			

^a^n (%); ^b^mean (SD); ^c^data were analyzed by ANOVA; ^d^data were analyzed by MANOVA.

**Table 2** presents the distribution of sex and the mean and SD of age, social communication deficits, inattention, hyperactivity/impulsivity, opposition, depression, anxiety, suicidality, and self-esteem among the groups of various self-reported bullying involvement. Because the data of suicidality was skewed, square root transformation is used to make the data of suicidality fit a normal distribution. The result of MANOVA revealed that there was a significant difference in depression, anxiety, suicidality, and self-esteem among groups classified based on self-reported bullying involvement. The further analysis indicated that the self-reported perpetrator-victims and pure victims had more severe depression and anxiety than the neutrals. Regarding suicidality and self-esteem, no significant group difference in suicidality could be identified in *post hoc* comparison.

**Table 3** presents the distribution of sex and the mean and SD of age, social communication deficits, inattention, hyperactivity/impulsivity, opposition, depression, anxiety, suicidality, and self-esteem among the groups of various parent-reported bullying involvement. The parent-reported perpetrator-victims, pure victims, and pure perpetrators had more severe social communication deficits than the neutrals; the parent-reported perpetrator-victims and pure victims had more severe inattention and hyperactivity/impulsivity than the neutrals; the parent-reported perpetrator-victims had more severe opposition than the neutrals. The result of MANOVA revealed that the difference in depression, anxiety, suicidality and self-esteem among the four groups classified based on parent-reported bullying involvement was also significant. The results for group comparisons showed the parent-reported perpetrator-victims had more severe depression and anxiety than the neutrals.

**Table 4** shows the results of MANCOVA comparing the levels of depression, anxiety, suicidality, and self-esteem among self-reported and parent-reported pure bullying victims, pure bullying perpetrators, bullying perpetrator-victims, and the neutrals after controlling for the effects of demographic data, social communication deficits, inattention, hyperactivity/impulsivity, and opposition. The results indicated that compared with the self-reported neutrals, the self-reported perpetrator-victims and pure victims had more severe depression and anxiety. However, no difference was found in depression and anxiety between the self-reported pure perpetrators and the neutrals or among the self-reported pure victims, pure perpetrators, and perpetrator-victims. No difference in suicidality and self-esteem was found among the four groups of various self-reported bullying involvement experiences. No difference in depression, suicidality, anxiety, and self-esteem was found among the four groups of various parent-reported bullying involvement experiences.

**Table 4:** Comparisons of depression, anxiety, suicidality, and self-esteem among the groups of various self-reported and parent-reported bullying involvement: multivariate analysis of covariance^a^.

	Self-reported bullying involvement			Parent-reported bullying involvement	
	*F*	*p*	*Post hoc* comparison	*F*	*p*
Depression	3.436	<0.001	Perpetrator-victims > Neutrals^b^; Pure victims > Neutrals^b^	1.278	0.228
Anxiety			Perpetrator-victims > Neutrals; Pure victims > Neutrals		
Suicidality					
Self-esteem					

a Controlling for the effects of sex, age, social communication deficits, inattention, hyperactivity/impulsivity, and opposition.

b Weight least square used to adjust unequal variance.

## Discussion

The present study conducted in adolescents with ASD found that the self-reported perpetrator-victims and pure victims had more severe depression and anxiety than the self-reported neutrals. No difference in depression, suicidality, anxiety, and self-esteem was found among the four groups of various parent-reported bullying involvement experiences.

The present study found that the rates of the self-reported neutrals, pure victims, pure perpetrators, and perpetrator-victims in adolescents with high-functioning ASD based on the C-SBEQ were 63.9%, 17.8%, 9.1%, and 9.1%, respectively. A previous study using the same questionnaire in the Taiwanese general adolescent population found that the rates of the neutrals, pure victims, pure perpetrators, and perpetrator-victims were 65.2%, 15.3%, 9.8%, and 9.8%, respectively (18). The results indicated that the rates of various types of bullying involvement in adolescents with high-functioning ASD were similar to those in general population. However, inconsistent with the results of a previous study on the general adolescent population (18), the present study found that only the self-reported pure victims and perpetrator-victims with ASD but not the pure perpetrators with ASD had poor mental health compared with those with ASD without any involvement in bullying. Although the severities of depression and anxiety of adolescents with high functioning ASD were not higher than those of the general Taiwanese adolescent population evaluated by the same measures (18), depression and anxiety are important mental health problems warranted intervention. The results of the present study evidenced that adolescents with high-functioning ASD who have experienced bullying victimization have more severe depression and anxiety than those who have never involved in bullying. We suggest that mental health problems are a clinical focus warranted routine survey in ASD adolescents who were the victims of bullying.

It is noteworthy that the self-reported perpetrator-victims but not the pure perpetrators had more severe depression and anxiety than the self-reported neutrals. The present study did not explore the motivation of the perpetrator-victims to perpetrate bullying. Although bullying is considered as an aggressive action in which someone intentionally inflicts injury or discomfort upon others (40), reactive aggression can be found in bullying victims to defend themselves accompanied by anger and retaliate against maltreatment (41, 42). The perpetrator-victims with ASD may perpetrate reactive aggression to defend themselves when they encounter provocation and trouble, which may result in anxiety simultaneously. Furthermore, proactive aggression that is only found in bullying perpetrators is a goal-directed, deliberate, offensive action implemented to achieve goals; it requires no stimulus, and may be characterized by pleasure or satisfaction (41, 42). The pure perpetrators with ASD may perpetrate bullying to others without regret and therefore experience less mental health problems.

In the present study conducted in adolescents with ASD, no significant difference was found in mental health problems between the perpetrator-victims and pure victims and between the perpetrator-victims and pure perpetrators. The results of the present study were not in accordance with those of the previous study on the general adolescent population in Taiwan, which found that perpetrator-victims had greater risks of mental health problems than pure victims and pure perpetrators (18). Perpetrator-victims have been considered to be the most vulnerable group, as they are at higher risks of suffering multiple psychopathologies than those with other experiences of bullying involvement (10). The results of the present study suggest that psychosocial processes in bullying involvement differ between perpetrator-victims with ASD and those without ASD. For example, deficits in theory of mind skills are the one of core psychopathologies in ASD. Theory of mind skills is the ability of individuals to attribute mental states to themselves and to others in order to explain and predict behavior (43). Because of the deficits in social awareness, adolescents with ASD may be less able to recognize the existence of bullying than adolescents without ASD (5). However, whether and how these differences in psychosocial processes influence the association between perpetration-victimization and mental health problems in adolescents with ASD warrants further study.

The present study found low agreement between self-reported and parent-reported bullying involvement of adolescents with ASD. Research on general adolescent population found that adolescents may have different interpretations of interacting behaviors with peers compared with their teachers and parents (44). Because of the core symptoms of ASD, adolescents with ASD may misinterpret bullying situations as non-bullying (45). The results of MANOVA revealed that the parent-reported perpetrator-victims had more severe depression and anxiety than the neutrals, though the differences became nonsignificant after controlling for the effects of demographic data and adolescents’ social communication deficits, ADHD, and opposition symptoms. The results of the present study indicated that mental health professionals should take both self-reported and parent-reported bullying involvement into consideration simultaneously when developing prevention and intervention programs for bullying involvement in adolescents with ASD.

The findings of the present study had several clinical implications. First, parents reported higher rates of pure bullying victimization and victimization-perpetration than those reported by the adolescents with ASD. The results indicated that the parents of the adolescents with ASD were more likely to detect and report the adolescents’ bullying victimization than did the adolescents themselves. Moreover, mental health problems were only found to be significantly associated with adolescent-reported bullying involvement, but not with parent-reported ones. The results indicated that mental health and educational professionals should not rely on information reported by the sole source. Both adolescents’ self-reported and parent-reported bullying involvement should be collected to detect bullying involvement and related mental health problems in adolescents with ASD. Second, the present study found that both pure victims and perpetrator-victims had more severe depression and anxiety than the neutrals. Based on the results of the present study, bullying victimization, depression and anxiety should be routinely surveyed in adolescents with high-functioning ASD. Research found that the treatment program focusing on enhancing theory of mind performance ability could reduce bullying involvement in children and adolescents with high-functioning ASD (46). Moreover, there are some models of psychosocial approaches proposed to treat depression in children and adolescents with ASD (47). These prevention and intervention programs should be provided to those who need. Third, although the validity of the C-SBEQ warrants further examination in adolescents with high-functioning ASD, the C-SBEQ or other self-reported instruments can be used to collect ASD adolescents’ subjective experiences of bullying involvement and provide the basis of intervention.

This study has several limitations. First, the causal relationships between bullying involvement and mental health problems in adolescents with ASD could not be concluded in this cross-sectional study. The temporal sequence of different levels and types of involvement in bullying and mental health problems warrant longitudinal studies to examine. For example, a longitudinal study revealed that internalizing behaviors at the age of 13 years predicted victimization experiences at the age of 15 years in adolescents with ASD (48). Second, the study participants were adolescents with high-functioning ASD who visited medical units for treatment or survey. Therefore, the results of this study might not be generalizable to all adolescents with ASD. Third, although we collected data on adolescents’ and parents’ reports on bullying involvement, we did not collect the reports from school teachers and peers. Fourth, we did not examine the influences of treatment that the adolescents have received on the association between bullying involvement and mental health problems. As mentioned before, there have been psychosocial approaches proposed to reduce bullying involvement (46) and treat depression in children and adolescents with ASD (47). Previous experiences of receiving intervention for bullying involvement and mental health problems may influences the association between bullying involvement and mental health problems. Fifth, Olweus (40) proposed that bullying is a negative or malicious behavior intended to harm or distress others. The present study inquired neither the intention of adolescents with high-functioning ASD nor the intention of their classmates to perpetrate aggressive behaviors. Given that the adolescents with high-functioning ASD may not figure out their own or classmates’ intention to perpetrate aggressive behaviors, the rates of bullying involvement in adolescents with high-functioning ASD found in the present study may be not the same as the studies using the definition of bullying proposed by Olweus. Further study warrants to examine the findings using different definitions of bullying involvement.

## Conclusion

The present study found that ASD adolescents who self-reported to be the pure bullying victims or perpetrator-victims have more severe depression and anxiety than those who self-reported no involvement in bullying. Mental health and educational professionals should collect information from both adolescents and their parents to detect bullying involvement, and they should consider comorbid mental health problems when assessing adolescents with ASD who are involved in bullying.

## Ethics Statement

This study was carried out in accordance with the recommendations of Institutional Review Board (IRB) of Kaohsiung Medical University with written informed consent from all subjects. All subjects gave written informed consent in accordance with the Declaration of Helsinki. The protocol was approved by the IRB of Kaohsiung Medical University.

## Author Contributions

Conceptualization: W-JC, C-FY. Data analysis: C-FY, P-WW. Funding acquisition: W-JC, C-FY. Draft writing: RH, H-FH, C-FY.

## Funding

This study was supported by the grant NSC 102-2628-B-037-007-MY3 awarded by the National Science Council, Taiwan. This work has been reported in the 7th World Congress on Attention Deficit and Hyperactivity Disorders in April, 2019, Lisbon, Portugal.

## Conflict of Interest

The authors declare that the research was conducted in the absence of any commercial or financial relationships that could be construed as a potential conflict of interest.
